# Intussusception after open Roux-en-Y gastric bypass managed with laparoscopic reduction

**DOI:** 10.1093/jscr/rjab339

**Published:** 2021-08-10

**Authors:** Jeffrey P Marcoe, Wai Yip Chau

**Affiliations:** Department of Surgery, Penn Medicine Princeton Health, Plainsboro, NJ, USA; Department of Surgery, Penn Medicine Princeton Health, Plainsboro, NJ, USA

## Abstract

Intussusception is a rare long-term complication following bariatric surgery. With unclear risk factors and a variable presentation, intussusception is often diagnosed in emergency departments on cross-sectional imaging. Due to the nature of the disease process, prompt involvement of a bariatric surgeon and operative intervention offers the best outcome. Here, we discuss two similar cases of jejunojejunal intussusception following open Roux-en-Y gastric bypass and abdominoplasty that were managed with operative reduction of the involved bowel.

## INTRODUCTION

The Roux-en-Y reconstruction was first introduced by Swiss surgeon, Dr. Cesar Roux, at the turn of the nineteenth century in order to reconstruct the gastrointestinal tract. Although it was first introduced as a means to bypass gastric outlet obstruction from peptic ulcer disease, it has since been adopted by several fields of gastrointestinal surgery including trauma, oncologic, general and bariatric surgery. With development of the open gastric bypass in the 1960s, the Roux-en-Y reconstruction was utilized to reconstruct the gastrointestinal tract in order to provide the malabsorption element in the bariatric procedure. Since the incorporation of the Roux-en-Y reconstruction into bariatric surgery, intussusception has been a rare complication. The incidence of small bowel intussusception after gastric bypass surgery has been estimated to be between 0.1 and 1.2% [1, 2]. Amongst Roux-en-Y gastric bypass patients, intussusception can happen at either of the two anastomoses. Retrograde intussusception of the roux limb can be observed at the gastrojejunal anastomosis on upper endoscopy and is seldom pathogenic. Intussusception can also occur at the jejunojejunal anastomosis in which case it tends to be more clinically significant. Here, we present two cases of intussusception at the jejunojejunal anastomosis several years after open Roux-en-Y gastric bypass.

## CASE SERIES

**Figure 1:**
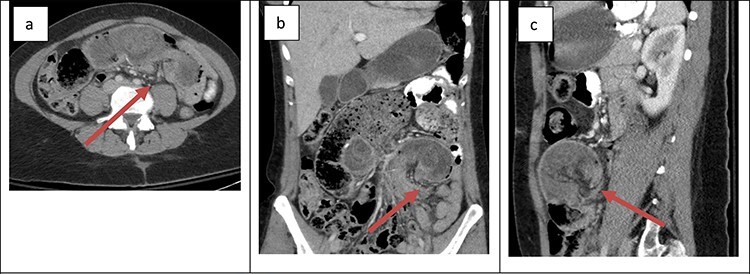
Cross-sectional imaging of displaying bowel and mesentery (arrow) intussuscepting into the JJ anastomosis on (**a**) transverse view, (**b**) coronal view and (**c**) sagittal view.

The first patient was a 36-year-old female with a history of type 1 plasminogen activator inhibitor deficiency, open Roux-en-Y gastric bypass 12 years prior, abdominoplasty with bilateral breast augmentation and chronic abdominal and back pain. She was 275 lbs at the time of her gastric bypass and 165 lbs at the time of presentation. The patient presented with a short history of new abdominal pain after a recent meal. No other associated symptoms were present upon presentation. Her vitals were within the normal limits and laboratory work was unremarkable. An abdominal ultrasound was obtained to evaluate for biliary disease, which revealed cholelithiasis without further evidence of acute process. Cross-sectional imaging revealed a small bowel obstruction secondary to small bowel intussusception at the jejunojejunal anastomosis (Fig. 1). The patient was taken emergently to the operating room for a laparoscopic lysis of adhesions and reduction of intussusception. In the operating room, ~60 cm of jejunum was found to be involved at the site of the jejunojejunostomy; after reduction, the involved bowel appeared viable so no formal resection or revision was performed. The patient’s post-operative course was uncomplicated. She was started on clear liquids on POD#1 and discharged home on POD#4. After discharge, the patient continued to experience episodic pain consistent with her chronic abdominal pain. Cross-sectional imaging ruled out further episodes of intussusception.

**Figure 2:**
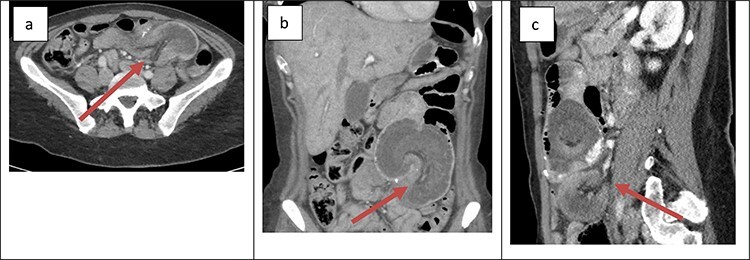
Cross-sectional imaging of displaying bowel and mesentery (arrow) intussuscepting into the JJ anastomosis on (**a**) transverse view, (**b**) coronal view and (**c**) sagittal view.

The second patient was a 41-year-old female with a history of open Roux-en-Y gastric bypass ~20 years prior and abdominoplasty with bilateral breast augmentation. Her only medication was Contrave to treat weight regain after bariatric surgery. She was 400 lbs at the time of her surgery and 185 lbs at time of presentation. The patient presented with an 8-h history of severe abdominal pain. The patient also reported two episodes of vomiting and two bowel movements prior to presentation. Her vitals were within the normal limits and laboratory work was unremarkable. Physical examination revealed a soft abdomen with a palpable mass at the site of tenderness in the left lower quadrant. Cross-sectional imaging revealed retrograde intussusception at the jejunojejunal anastomosis (Fig. 2); the cross-sectional imaging was also suggestive of hepatic vein thrombosis that was ruled out with a hepatic duplex ultrasound. The patient was taken emergently to the operating room for laparoscopic lysis of adhesions and reduction of intussusception. The involved small bowel measured ~30 cm in length and appeared grossly viable so no formal resection or revision was performed at the time of operation. The patient’s post-operative course was uncomplicated. She was started on clear liquids on POD#1 and discharged home on POD#2. The patient reported that she was symptom free at her 1-week post-operative office appointment.

## DISCUSSION

Jejunojejunal intussusception represents a rare complication after Roux-en-Y gastric bypass. The etiology of jejunojejunal intussusception is poorly understood. Several etiologies have been proposed and likely all contribute to its development. The patients presented above had lost 110 and 215 lbs, respectively from the time of their open gastric bypass to presentation. Thinning of the associated mesentery in these patients likely accommodates the laxity that allows for the small bowel to intussuscept into to anastomosis; in addition, the defunctionalized segment of bowel utilized to construct the anastomosis may promote intussusception due to the impaired motility [3]. The anastomotic staple line, the suture utilized to close the mesenteric defect or a segment of dysfunctional small bowel may act as the pathologic lead point that is classically present in cases of intussusception.

One study reviewing 20 years of data from 29 published studies worldwide to identify 71 patients with intussusception after Roux-en-Y gastric bypass reported that 98.6% of such patients were females, average time to presentation was 36 months after surgery and patients lost on average 145 lbs [3]. Another study reported that 50% of patients with intussusception after open gastric bypass had previously undergone an abdominoplasty and proposed that this may be a predisposing factor [4].

The presentation of intussusception is widely variable depending on the severity of the disease process. Common presentations include abdominal pain, nausea and vomiting, new palpable abdominal mass, lower GI bleeding and signs of sepsis. Intussusception can be diagnosed through various imaging modalities such as cross-sectional imaging, plain radiographs, small bowel series and ultrasonography. Cross-sectional imaging is believed to be not only highly sensitive but also reliable [3]. On cross-sectional imaging, intussusception appears as a complex soft tissue mass representing bowel telescoped onto itself often with an eccentric area of hypoattenuation within the mass representing the intussuscepted mesenteric fat [5]. The pathognomonic appearance is appreciated in Figs 1 and 2. Furthermore, the wall of the internalized loop of bowel can be observed within the dilated bowel when imaging is enhanced with intravenous contrast. Prompt diagnosis and surgical intervention are paramount in management of intussusception.

Several approaches (open versus laparoscopic) as well as methods of management of intussusception have been documented, including conservative management, reduction alone, reduction and enteropexy, and resection with revision of the anastomosis [2].

The patients presented above were similar in that both were female, had open gastric bypass surgery in their third decade of life, their surgery was greater than 10 years prior to presentation, they lost a significant amount of weight (>100 lbs) and underwent abdominoplasty. The first patient presented with only postprandial abdominal pain, whereas the second patient presented with nausea and vomiting. Cross-sectional imaging identified intussusception at the site of the jejunojejunal anastomosis in both patients. Interestingly, the patient whose only symptom was abdominal pain was obstructed secondarily to the intussusception and the patient who presented with nausea and vomiting did not have dilated loops of bowel proximal to the site of intussusception. Both cases were treated with emergency laparoscopy; the intussuscepted bowel was reduced without resection or enteropexy. Several factors led to this management approach including the long interval between the index bariatric surgery and presentation, the length of involved bowel (30+ cm), the viability of the small intestine after complete reduction and the absence of an obvious lead point. This approach also allows for future discussion with patients regarding an elective revision of the anastomosis once the edema subsides and they have been optimized.

In conclusion, intussusception following Roux-en-Y gastric bypass represents a rare long-term complication with poorly understood risk factors and etiology. Presentation can be variable with some component of abdominal pain, nausea and vomiting. There are several options to manage intussusception, selecting the method for a specific patient should take into consideration multiple factors including the patient’s overall clinical status and the condition of the bowel involved in the disease process.

## Conflict of interest statement

None declared.

## Funding

None.
